# Echovirus Type 7 Virus-Associated Hemophagocytic Syndrome in a Neonate Successfully Treated With Intravenous Immunoglobulin Therapy: A Case Report

**DOI:** 10.3389/fped.2019.00469

**Published:** 2019-11-14

**Authors:** Yuka Watanabe, Takahiro Sugiura, Mari Sugimoto, Yasuko Togawa, Masanori Kouwaki, Norihisa Koyama, Shinji Saitoh

**Affiliations:** ^1^Department of Pediatrics, Toyohashi Municipal Hospital, Toyohashi, Japan; ^2^Department of Pediatrics and Neonatology, Nagoya City University Graduate School of Medical Sciences, Nagoya, Japan

**Keywords:** hemophagocytic lymphohistiocytosis, newborn, intravenous immunoglobulin therapy, echovirus infections, vertical transmission

## Abstract

Virus-associated hemophagocytic syndrome (VAHS) in the neonatal period has a high mortality. Although clear diagnostic criteria and treatment methods have not been established, early diagnosis and treatment are critical. However, treatments for VAHS have potentially serious side effects, especially during the neonatal period. Echovirus type 7 can cause maternal infection around parturition and be vertically transmitted to the neonate and induce VAHS. Intravenous immunoglobulin (IVIG) therapy could be a first-line therapy for neonatal VAHS, so that treatments with potentially serious side effects, including cyclosporine A and etoposide, could be avoided. A case of VAHS associated with echovirus type 7 that was successfully treated with IVIG therapy is reported.

## Background

Virus-associated hemophagocytic syndrome (VAHS) is classified within acquired hemophagocytic lymphohistiocytosis (HLH) and is a rare and life-threatening disease resulting in pathological findings secondary to abnormal proliferation of activated lymphocytes and histiocytes leading to high levels of inflammatory cytokine release (1). Because neonatal HLH shows non-specific symptoms, it is difficult to distinguish it from other diseases. Diagnosis and treatment are often delayed, with mortality as high as 60%. Although the ideal therapy for patients with VAHS remains unknown, many of the therapies, such as immunosuppressant therapy, antitumor agent therapy, exchange blood transfusion, and blood purification methods, have potentially severe side effects. A case of severe VAHS in the neonatal period associated with echovirus type 7 that was successfully treated with intravenous immunoglobulin (IVIG) therapy is reported.

## Case Presentation

**Figure 1:**
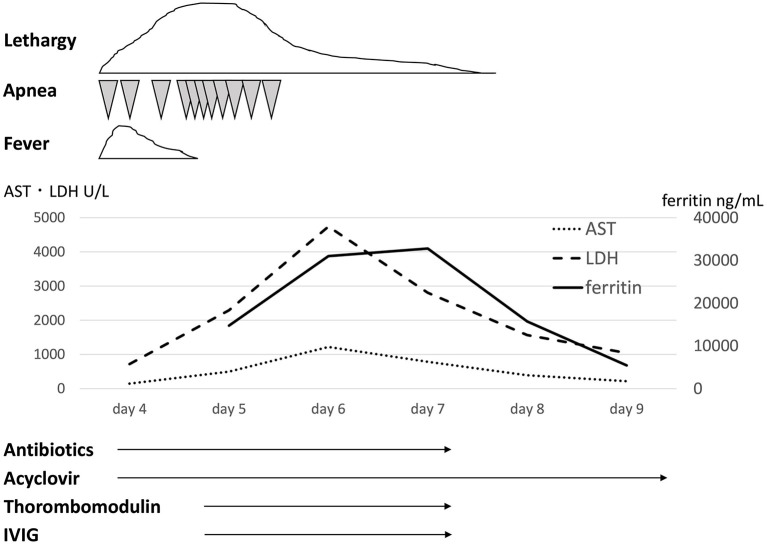
Clinical course and laboratory tests. AST, aspartate aminotransferase; ALT, alanine aminotransferase; IVIG, intravenous immunoglobulin.

A 37-week gestational age male infant was transferred to the neonatal intensive care unit (NICU) on the 4th day of life with apnea and lethargy. His two brothers had had fever and diarrhea 1 week before his birth. The maternal past medical history was unremarkable. At 37 weeks' gestation, his mother was suspected to have appendicitis due to fever and abdominal pain. She underwent cesarean section on that day, and she subsequently turned out not to have appendicitis, but to have a viral infection, presumably echovirus 7 infection. The infant was a boy with Apgar scores of 8 and 9 at 1 and 5 min, respectively. His birth weight was 3,190 g. He was in good condition and was in the nursery until 4 days of age. At 4 days of age, the infant was admitted to the NICU due to lethargy and apnea. His clinical course is shown in Figure 1. On admission to the NICU, he had frequent episodes of apnea that required ventilatory support. He did not have hepatosplenomegaly. Initial laboratory data demonstrated increased white blood cells (14,150/μL, 91% segmented neutrophils, 3% lymphocytes), aspartate aminotransferase (AST) (147 U/L), lactate dehydrogenase (LDH) (717 U/L), and C-reactive protein (1.86 mg/dL) levels. The cerebrospinal fluid examination showed a white blood cell count of 1,357/mm^3^, no red blood cells, 99.7% segmented neutrophils, 0.3% lymphocytes, protein 145 mg/dL, and glucose 61 mg/dL. The Gram stain was negative. There was no significant abnormality on thoracoabdominal X-ray examination. Treatment was started with ampicillin sodium (200 mg/kg/day), cefozopran hydrochloride (200 mg/kg/day), and acyclovir (60 mg/kg/day). At 5 days of age, the infant developed further frequent episodes of apnea, non-responsiveness to stimulation, and lethargy. The laboratory data showed rapid increases of LDH, AST, and ferritin levels. At 6 days of age, he developed disseminated intravascular coagulation although his physical condition, such as apnea attacks and lethargy, improved, and he was treated with fresh frozen plasma, platelet transfusion, and thrombomodulin at 5 and 6 days of age. The maximum values of LDH, AST, and ferritin were 4,750, 1,217 U/L, and 32,775 ng/mL, respectively. The minimum values of platelets and Hb were 21,000/μL and 9.9 g/dL, respectively. The laboratory data demonstrated hypofibrinogenemia (129 mg/dL) and a high soluble interleukin-2-receptor (sIL-2) (2,650 U/mL) level. He received 3 courses of IVIG (500 mg/kg, 1 g/kg, 500 mg/kg; total 2 g/kg) at 5, 6, and 7 days of age. After 7 days of age, his symptoms and laboratory findings improved. Echovirus 7 was demonstrated in cerebrospinal fluid, pharyngeal fluid, urine, and stool obtained within 5 days of age. He was finally diagnosed with VAHS associated with echovirus type 7. Viral tests of his mother and brothers were not performed. He recovered fully and was discharged home on the 23rd day of life. His development was normal at 1 year and 6 months of age.

## Discussion

HLH is usually diagnosed based on the HLH-2004 diagnostic criteria [Table 1; (2)]. Five or more of eight criteria must be fulfilled unless there is a family history or molecular diagnosis consistent with HLH. In the present case, 5 criteria were fulfilled (fever, bi-cytopenia, hypofibrinogenemia, hyperferritinemia, and high sIL2-R), and the patient was diagnosed with VAHS associated with echovirus type 7, presumably caused by vertical infection from the mother. The limitation of this case is that hemophagocytosis was not proven. VAHS in the neonatal period rarely satisfies the diagnostic criteria because of the metabolism peculiar to the newborn. For example, a high triglyceride level is rare. NK cell activity is not useful as a diagnostic criterion because the reference value in the neonatal period is unclear. Furthermore, fever, which is one of the main symptoms of HLH, is not frequent, because the temperature of premature neonates is controlled by adjustments in the ambient air temperature.

**Table 1:** Diagnostic guidelines for HLH (HLH-2004 criteria).

**The diagnosis of HLH can be established if either A or B is fulfilled**.
A. Molecular diagnosis consistent with HLH
B. Diagnostic criteria for HLH fulfilled (five of the eight criteria below)
**1. Fever**
2. Splenomegaly
**3. Cytopenias (affecting ≥two of three lineages in the peripheral blood):**
**Hemoglobin <9 g/dL (in infants <4 weeks: hemoglobin <10 g/dL)**
**Platelets <100,000/μL**
**Neutrophils <1,000/ μL**
**4. Hypertriglyceridemia and/or hypofibrinogenemia:**
**Fasting triglycerides ≥265 mg/dL**
**Fibrinogen ≤150 mg/dL**
5. Hemophagocytosis in bone marrow or spleen or lymph nodes
**No evidence of malignancy**
6. Low or absent NK cell activity
**7. Ferritin ≥500 ng/mL**
**8. Soluble IL-2 receptor ≥2400 U/mL**

*The five criteria satisfied in this case report are shown in bold letters. HLH, hemophagocytic lymphohistiocytosis; NK, natural killer*.

In neonates, VAHS associated with infection with non-EBV, including enterovirus, parechovirus, and cytomegalovirus, is the major cause. Echovirus type 7 is a virus belonging to the enterovirus genus. Echovirus infection is known to show various clinical manifestations in children and adults. They include upper respiratory tract symptoms, diarrhea, rash, hepatitis, abdominal pain, myocarditis, meningitis, and inapparent infection. Echovirus type 7 infections have also been reported in neonates, with higher mortality than in adults. There have been five reports of suspected VAHS associated with echovirus type 7 in the neonatal period; four patients died, and one survived [Table 2; (3–7)]. According to these reports, echovirus type 7-associated neonatal VAHS clearly shows a poor prognosis. Although factors for predicting the prognosis of VAHS in the neonatal period are unclear, several indicators have been suggested. The tissue invasiveness of viruses can be an index for defining prognosis. There are other factors related to prognosis, for example, the amount of virus, vertical infection, the amount of transplacental-derived antibody, and immunity of neonates. Gestational age may be one of the factors. All neonates who died from VAHS associated with echovirus type 7 were preterm infants. Vertical infection seems to be particularly important. Transplacental viremia is thought to cause VAHS in a neonate by infecting the fetus with a large amount of virus. It is also suggested that the amount of maternal antibody is related to the onset and severity of VAHS. However, it is difficult to identify the type of virus and the amount of maternal antibody as predictors in clinical practice, but there is a classification to determine the severity based on laboratory findings [Table 3; (8)]. With this classification, the present patient was classified as severe. He could have had transplacental viremia and a small amount of maternal antibody against echovirus type 7 because cesarean section was done on the day of his mother's onset of fever.

**Table 2:** Neonatal cases suspected of VAHS associated with echovirus 7.

**Cases**	**Gestational age (weeks)**	**Birth weight (g)**	**Route of infection**	**Therapy**	**Endpoint**
Kazzi et al. (3)	33	1,610	Vertical infection	Antibiotic Blood exchange Transfusion IVIG	Death
Wreghitt et al. (4)	31	1,980	Vertical infection	Antibiotic Blood exchange Transfusion	Death
Castro (5)	28	1,101		Antibiotic IVIG	Death
Tancabelic and Haun (6)	36	2,300	Vertical infection	Antibiotic Acyclovir Transfusion rFVIIa	Alive
Rentz et al. (7)	35		Vertical infection	Antibiotic IVIG Antiviral drug Serum transfusion of infected mother	Death

**Table 3:** Scoring of HLH.

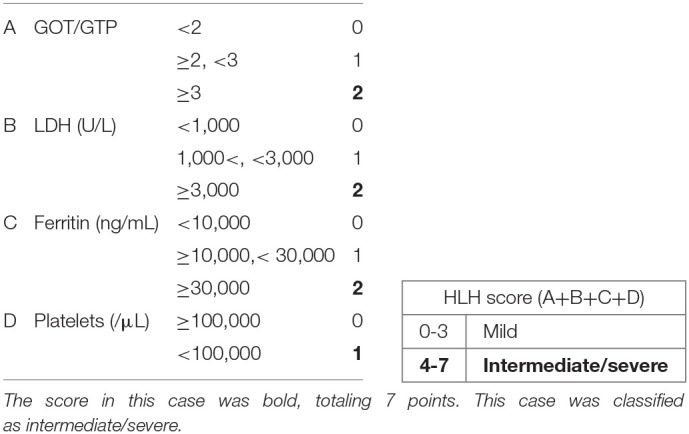

*The score in this case was bold, totaling 7 points. This case was classified as intermediate/severe*.

The ideal therapy for neonates with VAHS remains unknown. Treatments include IVIG, steroids, cyclosporine A, etoposide, exchange blood transfusions, and blood purification methods. Serious side effects and complications are expected with these treatments. Immunosuppressants and anticancer drugs are particularly rarely used for neonates due to severe side effects such as bone marrow suppression, kidney damage, and secondary cancer. On the other hand, there have been some reports of VAHS in the neonatal period treated by IVIG. Three of five cases of VAHS in the neonatal period caused by echovirus type 7 were treated by IVIG (3–7). High-dose intravenous immunoglobulin is used currently for the treatment of autoimmune or inflammatory diseases. Although the mechanism has not been clarified, it has been proposed that suppression of cytokine networks, modulation of the complement cascade, and regulation of cell proliferation may be involved (9). A dose of 1–2 g/kg of intravenous immunoglobulin is commonly used for inflammatory diseases, such as Kawasaki disease and acute disseminated encephalomyelitis (10, 11). It seems reasonable to use IVIG for not only infections, but also abnormal proliferation of activated lymphocytes caused by infection. It is of note that the patient recovered with IVIG treatment alone. The reason for the successful treatment may be due to the early administration of IVIG. One patient who died from VAHS associated with echovirus type 7 was treated by IVIG 6 days after onset (7), and another was treated by IVIG after several intensive treatments including blood exchange transfusion (3). It would be important to control viral growth and inflammation in the early phase. Therefore, IVIG therapy in the early phase could be a first-line therapy for neonatal VAHS, so that treatments with potentially serious side effects, including cyclosporine A and etoposide, could be avoided.

In conclusion, a neonate with echovirus 7-associated VAHS was successfully treated with IVIG. IVIG therapy could be a first-line therapy for neonatal VAHS because of its relative safety. Further cases need to be evaluated to establish the standard management of neonatal VAHS.

## Concluding Remarks

Echovirus type 7 can cause maternal infection around parturition and be vertically transmitted to the neonate and induce VAHS. A case of a neonate with echovirus 7-associated VAHS that was successfully treated with IVIG was described. IVIG therapy could be a first-line therapy for neonatal VAHS, so that treatments with potentially serious side effects, including cyclosporine A and etoposide, could be avoided.

## Data Availability Statement

All datasets generated for this study are included in the article/supplementary material.

## Ethics Statement

The studies involving human participants were reviewed and approved by Toyohashi Municipal Hospital. Written informed consent to participate in this study was provided by the participants' legal guardian/next of kin.

## Author Contributions

YW cared for the patient, collected samples, and drafted the manuscript. TS cared for the patient, interpreted the patient data, and critically revised the manuscript. MS and YT cared for the patient and collected samples. MK cared for the patient and interpreted the patient data. NK and SS interpreted the patient data and critically revised the manuscript. All authors have read and approved the final manuscript.

### Conflict of Interest

The authors declare that the research was conducted in the absence of any commercial or financial relationships that could be construed as a potential conflict of interest.

## References

[1] Woods CW Bradshaw WT Woods AG Hemophagocytic lymphohistiocytosis in the premature neonate. Adv Neonatal Care. (2009) 9:265–73. 10.1097/ANC.0b013e3181c20010 20010142

[2] Henter JI Horne A Arico M Egeler RM Filipovich AH Imashuku S. HLH-2004: diagnostic and therapeutic guidelines for hemophagocytic lymphohistiocytosis. Pediatric Blood Cancer. (2007) 48:124–31. 10.1002/pbc.21039 16937360

[3] Kazzi NJ Cepeda EE Budev H. Fatal echovirus type 7 in a premature infant. Amer J Perinatol. (1988) 5:236–8. 10.1055/s-2007-999693 3382482

[4] Wreghitt TG Sutehall GM King A Gandy GM. Fatal echovirus 7 infection during an outbreak in a special care baby unit. J Infect. (1989) 19:229–36. 10.1016/S0163-4453(89)90709-3 2600441

[5] Castro R. Echovirus 7 infection and necrotizing enterocolitis-like symptoms in a premature infant. J Perinatol. (2000) 20(8 Pt 1):558–61. 10.1038/sj.jp.7200467 11190599

[6] Tancabelic J Haun SE. Management of coagulopathy with recombinant factor VIIa in a neonate with echovirus type 7. Pediatric Blood Cancer. (2004) 43:170–6. 10.1002/pbc.20078 15236286

[7] Rentz AC Libbey JE Fujinami RS Whitby FG Byington CL. Investigation of treatment failure in neonatal echovirus 7 infection. Pediatric Infect Dis J. (2006) 25:259–62. 10.1097/01.inf.0000202071.38484.93 16511392

[8] Kawa K Sakata A Takeshita Y Inoue M. [Diagnosis and treatment of hemophagocytic syndrome]. [Rinsho ketsueki] Jap J Clin Hematol. (2005) 46:418–23. 16447723

[9] Durandy A Kaveri SV Kuijpers TW Basta M Miescher S Ravetch JV. Intravenous immunoglobulins–understanding properties and mechanisms. Clin Exp Immunol. (2009) 158(Suppl. 1):2–13. 10.1111/j.1365-2249.2009.04022.x 19883419 PMC2801035

[10] McCrindle BW Rowley AH Newburger JW Burns JC Bolger AF Gewitz M. Diagnosis, treatment, and long-term management of Kawasaki disease: a scientific statement for health professionals from the American Heart Association. Circulation. (2017) 135:e927–9. 10.1161/CIR.0000000000000484 28356445

[11] Sahlas DJ Miller SP Guerin M Veilleux M Francis G. Treatment of acute disseminated encephalomyelitis with intravenous immunoglobulin. Neurology. (2000) 54:1370–2. 10.1212/WNL.54.6.1370 10746613

